# The Influence of Socio-Economic Factors on Diet and Active Lifestyle in the Spanish Female Population

**DOI:** 10.3390/nu15153319

**Published:** 2023-07-26

**Authors:** Elena Sandri, Eva Cantín Larumbe, Germán Cerdá Olmedo

**Affiliations:** 1 Faculty of Medicine and Health Sciences, Catholic University of Valencia San Vicente Mártir, c/Quevedo 2, 46001 Valencia, Spain; german.cerda@ucv.es; 2 Doctoral School, Catholic University of Valencia San Vicente Mártir, c/Quevedo 2, 46001 Valencia, Spain; 3 Escuela Técnica Superior de Ingeniería Informática, Polytechnical University of Valencia, Camí de Vera s/n, 46022 Valencia, Spain; ecanlar@etsinf.upv.es

**Keywords:** women, healthy eating index, socio-economic factors, physical activity, sedentary lifestyle

## Abstract

A balanced diet and healthy social habits are two pillars on which the health of the population is based. Therefore, the efforts of the health system should be aimed at prevention. To this end, it is important to know the prevalence of these habits in different population groups and how they vary according to socioeconomic variables. This is an observational, descriptive, cross-sectional study using surveys. A questionnaire was designed to explore a set of variables related to diet and an active lifestyle and was validated through a pilot study and a nominal group. Dissemination was carried out online through social networks by means of non-probabilistic snowball sampling, obtaining a sample of 14.784 women aged between 18 and 45 years. Bivariate comparative analyses were performed using the Mann–Whitney method and the principal component analysis (PCA) method of dimensionality reduction was used to study the relationships between ordinal numerical variables. Results indicate that nutrition was influenced by the age of the sample; adult women have better nutrition than younger women, although they are more sedentary and do less sport. Women with higher education and a medium-high income have better nutrition and healthier lifestyles and a lower BMI and higher self-perceived health status than women with basic education and a lower income. It was concluded that a higher level of income and a higher level of education generally lead to a healthier lifestyle. Spanish women aged 18–45 years need to make changes in their nutrition and lead a more active life.

## 1. Introduction

The importance of an adequate diet is becoming increasingly evident in society, as are the negative consequences of an incorrect diet, such as cardiovascular diseases [1], some cancers [2] or obesity [3], among others.

There is a growing number of studies on diet quality indicators related to food groups [4,5] and different indices have been developed to measure diet quality or the degree of adherence to a given dietary pattern [6].

The frequency of consuming different types of foods can have varying effects on health. While fruits and vegetables are generally considered beneficial and should be consumed frequently [7,8], fried and ultra-processed foods, for example, can have negative consequences when consumed too often [9,10,11]. It is important to maintain a balanced diet that includes a variety of nutrient-dense foods while limiting the intake of fried and ultra-processed foods. Moderation is the key, and it is generally recommended to reserve these types of foods for occasional indulgences rather than making them a regular part of your everyday diet. Prioritizing whole, unprocessed foods such as fruits, vegetables, whole grains, lean proteins, and healthy fats is crucial for maintaining good health.

But it is not only diet that influences the health of the population, and there are different studies [12,13,14] that show that there is a relationship between the practice of healthy social and sporting habits and an increase in the quality of life of the population, as well as a lower prevalence of diseases.

Sedentary lifestyles and the practice of sports have significant impacts on the health of the population. Sedentary lifestyles, characterized by a lack of physical activity and prolonged periods of sitting or inactivity, have been associated with a higher risk of various health problems [15]. Insufficient physical activity can contribute to weight gain, obesity, cardiovascular diseases, type 2 diabetes, certain cancers, and musculoskeletal issues [16]. Additionally, a sedentary lifestyle can have negative effects on mental health, including an increased risk of depression and anxiety [17,18].

On the other hand, engaging in regular physical activity and participating in sports offer numerous health benefits [19]. Physical activity helps to maintain a healthy body weight [20], improve cardiovascular health [21], strengthen muscles and bones, enhance flexibility and balance, and boost overall physical fitness. It also plays a crucial role in reducing the risk of chronic diseases such as heart disease, stroke, type 2 diabetes, certain cancers, and depression [22]. Regular exercise has been shown to improve cognitive function and promote better sleep patterns [23].

Given all of the above, it is therefore clear that it is important to know the social and nutritional habits of the population to define public policies and design training and information actions to contribute to making these habits healthy. Understanding them also gives us a very real idea of the state of health of the population, hence the growing importance currently given to this type of study.

There exist several studies in Spain that investigate lifestyle and nutrition among the population [6,24,25,26,27]. However, if there is a limited focus on studies specifically targeting the female population, it could be an area where more research is needed.

Women are a determining factor in the health management of the family and the community [28,29] and in recent decades they have been confronted with significant changes in their lifestyles due to the socio-cultural and economic changes that society has undergone. These changes have sometimes had consequences for their health and well-being, such as, for example, the increasingly frequent incorporation of women into the labor market, which has generated situations of physical and mental overload due to the effort to reconcile work and family life.

Women also experience unique hormonal changes during their lives [30], with two particularly significant moments being pregnancy [31] and menopause, which can have important repercussions for their health [32]. This is why women need specific care and support during the different stages of life. Women’s health needs are diverse and may vary from person to person, as are their dietary and nutrient needs. 

Understanding the unique aspects of women’s lifestyles and nutritional needs is important, as they can differ from those of men due to various factors such as physiological differences, reproductive health, and social roles. Investigating women’s lifestyles and nutrition can provide valuable insights into their overall health, well-being, and the specific health issues that may affect them. 

For all the above reasons, it seems particularly interesting to focus on the female figure when studying her lifestyle and health and the relationship with different environmental variables.

Finally, the last aspect that brings novelty to the present research is linked to the instrument used. There are several questionnaires in the literature that focus on studying eating habits and nutrition [25,33,34,35,36].

However, instruments to study lifestyle habits in a more holistic way are scarce. This is because lifestyle habits are very broad and comprise a variety of multidimensional and multifactorial aspects that are difficult to measure. 

This is why we consider that another contribution of this research is the development of an instrument that, without claiming to be exhaustive, allows different aspects of lifestyle to be measured in a more unified way. An instrument that, thanks to the fact that it can be self-completed and disseminated telematically through different channels, has the strength of being able to collect from a very broad sample of the population.

## 2. Materials and Methods

### 2.1. Subjects

A prospective cross-sectional study was carried out with data obtained on the Spanish adult female population residing in Spain, in the age range of 18 to 45 years, both included.

The inclusion criteria used in the study were:

− population between 18 and 45 years of age
− of Spanish nationality
− resident in Spain
− of female sex

The exclusion criteria were:

− respondents who had a chronic illness that could affect their diet.
− respondents who, at the time of the survey, were in a situation that temporarily deregulated their usual diet: hospitalization, prison admission, etc.

### 2.2. Ethical Approval

This study was conducted according to the guidelines laid down in the Declaration of Helsinki and all procedures involving human subjects were approved by the Research Ethics Committee of the Catholic University of Valencia, number of project UCV/2019-2020/152. Written informed consent was obtained from all subjects/patients.

### 2.3. Instruments and Variables

For data collection, a questionnaire was designed consisting of 53 self-designed questions, some of which were qualitative and others quantitative, to gather information on different descriptive variables of the sample (Table 1). 

Some variables were collected in a qualitative format, giving the option of choosing one among multiple options, as is the case, for example, of: sex, place of birth and residence (where a list of all the provinces of Spain was presented), level of education (where a choice was given between all the possible levels of education in the educational system), level of income (where salary steps were presented), usual residence (where a list of possibilities was presented) and all the frequencies of food and drink consumption. Other variables had a continuous quantitative value such as age, weight, and height (which were self-reported values) and others had a discrete quantitative value in a Likert scale format such as self-reported level of health, quality of sleep or feeling of waking up feeling rested.

For the validation of the questionnaire, a pilot survey was firstly prepared and given to a group of 53 people. Subsequently, a nominal group of seven experts in areas related to the research topic was convened so that, after the analysis and discussion of the survey provided to the pilot group, they could help to draw up the final questionnaire. Specifically, the expert group was made up of two psychologists, a nutritionist, a social educator, two family doctors, and a marketing and communication professional. As a result of this meeting, the final questionnaire was obtained, which presents content validity by means of qualitative-quantitative triangulation.

### 2.4. Data Collection

The questionnaire was designed for telematic transmission using the Google Form program so that it would be available on the internet and its dissemination would be quick and easy.

The questionnaire was distributed by non-probabilistic snowball sampling and was available for online response between August 2020 and November 2021.

The survey was disseminated using different media:

1. Personal social networks (LinkedIn, Twitter, Facebook).
2. Chain mails and WhatsApp messages.
3. Mailing to different establishments throughout Spain, selected because of the heterogeneous public (pharmacies, clinics, etc.).
4. An Instagram account @elretonutricional was created and used specifically to disseminate the survey, from which several professionals and influencers were contacted.

### 2.5. Healthy Nutrition Index

For the calculation of the healthy nutrition index, a reduced version of the IASE: Índice Alimentación Saludable para la población Española (Healthy Eating Index for the Spanish population) validated in the work of Norte and Ortiz [37] was used.

The first four variables of the indicator represent the food groups of daily consumption (Fruit, Vegetables, Cereals and Milk and derivatives), the fifth and sixth correspond to the food groups of weekly consumption (Meat and Pulses), where to calculate the meat consumption the mean between the consumption of white meat and red meat was taken. The seventh item, soft drinks, coincides with the food group of occasional consumption and finally the last variable considers the variety of the diet which is a necessary element in a healthy diet.

The scoring criteria applied for each of the variables based on the responses obtained in the survey follow the same dynamic as the IASE scores. The maximum score for each item is 10, which means that the recommendations proposed by the Spanish Society of Community Nutrition (SENC) are met for that variable [38]. The maximum obtainable score is 73.

### 2.6. Statistical Analysis

The information obtained was systematized in Excel and SPSS v.23 (SPSS Inc., Chicago, IL, USA) with the necessary preparation and coding of the variables for subsequent statistical analysis. Discrete variables are shown as absolute values and percentages. Continuous variables are shown as mean and standard deviation.

Firstly, a study of the normality of all the variables was carried out using the Kolmogorov–Smirnov test with Lilliefors correction, determining that none of the variables studied complied with normality, as is usual in large samples. Thus, for the comparison of means and the study of significance, we used the non-parametric Mann–Whitney U test for independent samples with a significance level of 0.05.

To study the relationships between ordinal numerical variables, without carrying out analyses for each pair of variables, the principal component analysis (PCA) method was used to reduce to dimensionality. Two principal components were selected to facilitate interpretation. 

**Figure 1:**
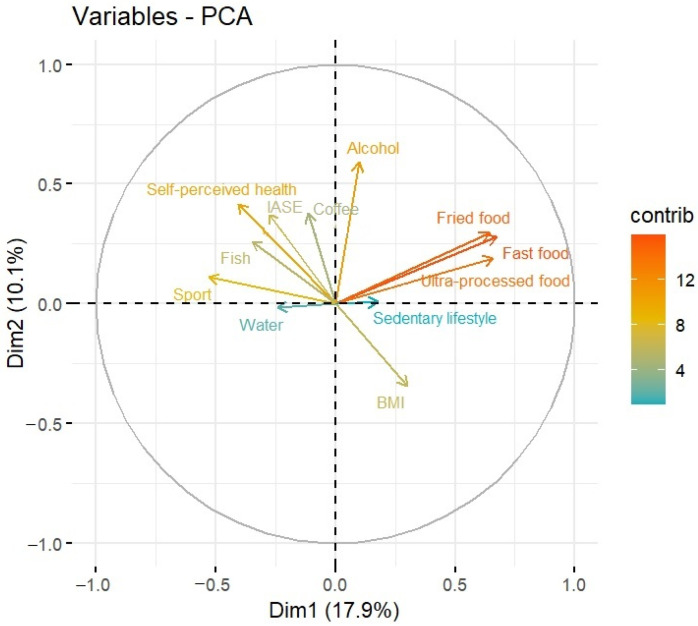
Graphical representation of the PCA models in 2 dimensions.

**Figure A1:**
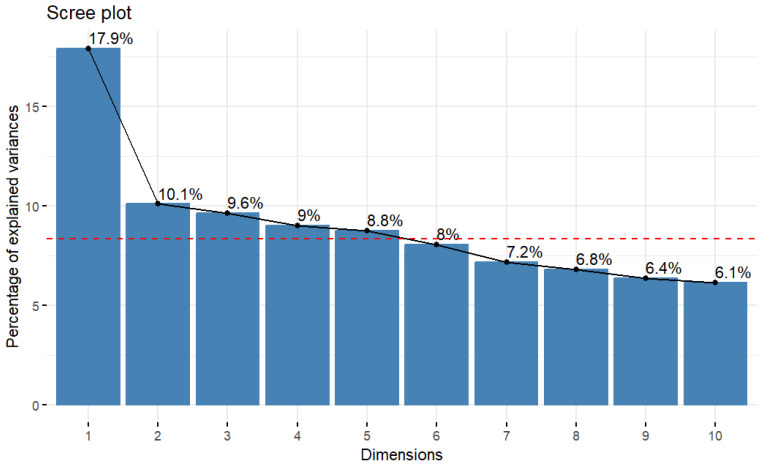
Graph of the participation of each dimension in the model. (NOTE: The red line indicates the average percentage of explained variance if all dimensions explain the same.)

**Figure A2:**
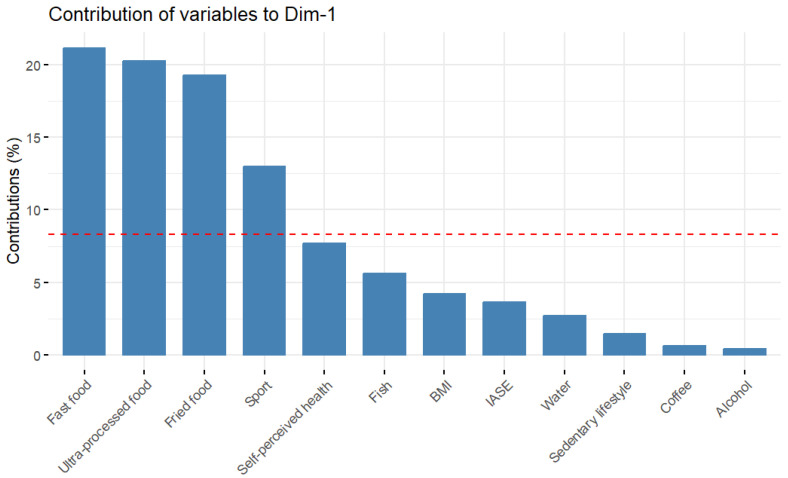
Graph of the contribution of the variables of dimension 1 of the model. (NOTE: The red line indicates the average percentage of explained variance if all dimensions explain the same.)

**Figure A3:**
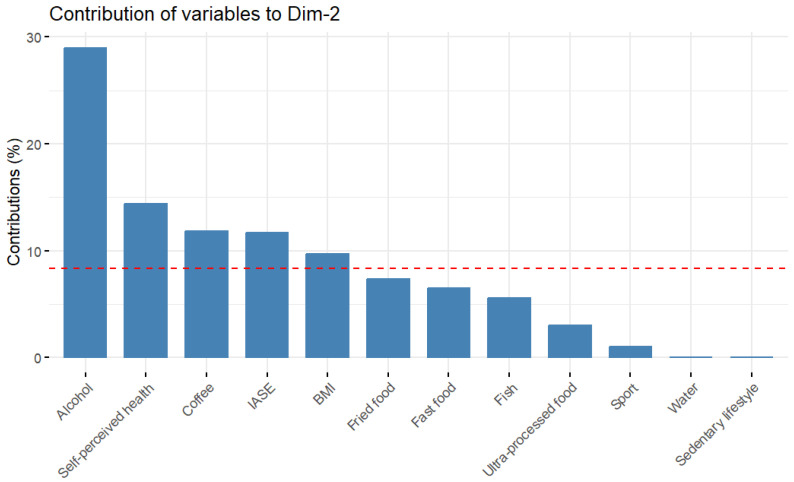
Graph of the contribution of the variables of dimension 2 of the model. (NOTE: The red line indicates the average percentage of explained variance if all dimensions explain the same.)

Two principal components were chosen regarding the scree plot: as can be seen in Figure A1 in Appendix A, the percentage variability explained by a new principal component is almost the same as the previous one. In addition, a PCA model’s interpretability is more straightforward, considering two dimensions rather than three. Figure A2 in Appendix A shows the graph of the contribution of the variables to dimension 1 of the model. The first dimension is mainly explained by Fast Food, Ultra-processed Food, Fried Food, and Sport. This also can be observed in Figure 1 in the length of the arrows, the proximity of these arrows to the *X*-axis, and their color, which is more reddish than blueish. On the other hand, Figure A3 in Appendix A shows the graph of the contribution of the variables to dimension 2 of the model, and show how Alcohol, Self-perceived health, Coffee, IASE, and BMI are the variables that contribute the most to the second dimension. This contribution can be seen in Figure 1, regarding the proximity of these rows to the *Y*-axis, more than the X one, and the length of the arrows, which are longer than the other ones. This method was carried out with RStudio 4.3.0.

## 3. Results

A total of 22.205 people of all ages and residing in all parts of Spain, including the Canary Islands and naturalized foreigners, answered the survey. Surveys that did not meet the inclusion criteria and those with invalid or incomplete data were discarded. It was finally decided to limit the sample to those aged between 18 and 45, to minimize the possible bias caused by the greater difficulty in accessing the internet and social networks that most of the older population has.

The sample obtained has the following characteristics:

− Sample size: 14.784 valid surveys.
− Age: 53.49% (7.908 people) aged between 18 and 30 years old and 46.51% aged between 31 and 45 years old (6.876 people). Mean = 30.41, Mode = 23, Median = 30.
− Level of studies: 30.56% (4.518) with basic education (No studies, Primary school, Vocational training or Secondary School) and 69.44% (10.266 respondents) with higher education (Degree, Master’s, or Doctorate).
− Income level: 51.86% (6.974 respondents) with a low purchasing power (less than EUR 2200 per month for the family nucleus) and 48.14% (6.473 people) with a medium-high purchasing power.

The behavior of the sample was studied concerning body mass index (BMI), IASE, and other health and behavioral variables, distinguished by age, educational level, and income level (Table 2).

Data indicate that the BMI of young people is significantly lower than that of adults (22.78 versus 24.06); young people are also more physically active (148 min per week versus 128 min) and consume less coffee (1.59 versus 1.75). In contrast, other lifestyle habits are less healthy than those of the adult population: IASE is lower (53.19 versus 54.43), sedentary behavior is higher (1.65 versus 1.53), the frequency of alcohol consumption is higher, as is the frequency of fast food, fried food and ultra-processed food consumption, while the frequency of fish consumption is lower. In line with this health trend is the level of self-perceived health, which is lower among young people than among adult women.

Women with basic education have a higher BMI (24 versus 23.15), a worse healthy eating index (51.95 versus 54.56), and a worse self-perceived level of health (3.65 versus 3.85). They also do less sport and consume alcohol, fried food and ultra-processed food more frequently while their frequency of fish consumption is lower. On the other hand, subjects with lower education consume alcohol and coffee less frequently and spend less time sitting down.

Finally, women with a lower income level have a higher BMI (23.65 versus 23.13), a worse score in the IASE (52.85 versus 54.81), and a worse level of self-perceived health (3.69 versus 3.90). They also have a higher frequency of fast food, fried food and ultra-processed food consumption while their frequency of fish consumption is lower. In contrast, people with lower incomes seem to have a less sedentary lifestyle (1.54 versus 1.64), spend more time per week in sports (142 min per week versus 134 min) and seem to consume alcohol less frequently than women with higher incomes.

Regarding the PCA analysis, two dimensions (principal components) are chosen which explain 28% of the variability of the data. The PCA graph obtained is represented in Figure 1, where, following the color scale in the legend, the variables colored in red and orange (fast food, fried food, and ultra-processed food) contribute the most to the model, while those colored in blue (sedentary lifestyle and water consumption) contribute the least. The contribution of the other variables studied is medium, although the frequency of alcohol consumption, self-perceived health, and time spent in sport (shown in yellow) contribute slightly more to the model than the variables frequency of fish and coffee consumption, IASE and BMI (shown in olive green).

## 4. Discussion

The statistical data obtained allow us to conclude that the behavior regarding health-related social habits and type of nutrition in the Spanish female population is influenced by age. Adult women between 31 and 45 years of age have a better healthy nutrition index than younger women, which is also supported by a lower frequency of consumption of fast food and fried or ultra-processed foods. Extensive research has shown that frequent consumption of fast food, fried foods, and ultra-processed foods can have detrimental effects on health [39,40]. These types of foods are often high in unhealthy fats, added sugars, sodium, and artificial additives while being low in essential nutrients [41].

The regular intake of fast food, which is typically high in calories, unhealthy fats, and sodium, has been associated with an increased risk of obesity, type 2 diabetes, heart disease, and certain types of cancer. Fast food meals often lack the necessary vitamins, minerals, and dietary fiber that are important for maintaining optimal health [42,43,44].

Fried foods, especially those prepared using unhealthy oils, can be particularly harmful to health. They are often high in trans fats, saturated fats, and calories. Frequent consumption of fried foods has been linked to an increased risk of obesity, high blood pressure, unhealthy cholesterol levels, and cardiovascular diseases [45,46].

Ultra-processed foods, which undergo extensive industrial processing and contain numerous additives, have become increasingly prevalent in modern diets. These foods are typically high in refined carbohydrates, unhealthy fats, added sugars, and artificial additives. Frequent consumption of ultra-processed foods has been associated with weight gain, metabolic disorders, increased inflammation, and a higher risk of chronic diseases such as obesity, type 2 diabetes, cardiovascular diseases, and certain types of cancer [9,40].

In the same health trend, it is observed that the adult population consumes fish more frequently. Frequent consumption of fish can have several health benefits [47]. Fish is a rich source of high-quality protein, vitamins, and minerals. It is also an excellent source of omega-3 fatty acids that help reduce the risk of heart disease [47]. Omega-3 fatty acids are also crucial for brain health and development. They are associated with improved cognitive function and memory and may help reduce the risk of age-related cognitive decline and neurological disorders such as dementia [48]. Regular fish consumption has also been linked to a reduced risk of age-related macular degeneration, a major cause of vision loss in older adults [49]. Finally, some studies suggest that regular fish consumption may be associated with a reduced risk of depression and with improved mental well-being. Omega-3 fatty acids are involved in brain function and are thought to have mood-stabilizing effects [50]. To prioritize health, it is important to reduce the intake of fast food, fried foods, and ultra-processed foods.

These healthier nutrition habits may translate into better body sensation, as adult women also reported better self-perceived health scores than younger women.

On the other hand, and to the detriment of health, it has been found that adult women had a more sedentary lifestyle, spending more hours a day sitting down and spending less time per week on physical activity than younger women. This lower physical activity, together with the natural increase in weight with age [51,52], could explain the higher BMI among adult women compared to younger women.

Adult women, especially as they get older, tend to lead a more sedentary lifestyle compared to younger women [53,54]. This is of concern because sedentary behavior and insufficient physical activity can have negative health consequences.

As women age, various factors such as work demands, caregiving responsibilities, and lifestyle changes can contribute to increased sedentary behavior. These factors can lead to increased sitting and activities that involve minimal physical movement. 

Reduced physical activity and increased sedentary behavior among adult women may contribute to an increased risk of weight gain, obesity, cardiovascular disease, type 2 diabetes, musculoskeletal problems, and general decline in functional abilities [15,16,17,18]. 

It is crucial to address these challenges and promote physical activity among adult women. Encouraging regular exercise and reducing sedentary behavior can have important benefits for health and well-being. This may include incorporating activities such as walking, cycling, swimming, strength training, or flexibility exercises into daily routines.

It is also essential to create environments and opportunities that are conducive to physical activity. This may involve community initiatives, workplace wellness programs, and accessible exercise options. In addition, education and awareness campaigns can help highlight the importance of physical activity for women’s health and provide information on ways to overcome barriers.

The fact that statistically significant differences were found for BMI and physical activity values between women with basic education and women with higher education is in line with other studies in the literature that have shown that the level of education has an important influence on obesity in Spanish women [55]. These data provide an important field of intervention in terms of prevention and training policies given that an incorrect diet, with a high fat content, together with a reduction in physical activity, according to the World Health Organization (WHO), are the main causes of the obesity pandemic that Western countries are currently experiencing.

The rate of healthy nutrition is also significantly higher among women with higher education compared to those with basic education. This health trend is also supported by the frequency of consumption of fast food and fried and ultra-processed foods, which is found to be lower in women with higher education and by the frequency of fish consumption, which is higher. The data are in line with previous studies that associate better education and higher educational attainment with better nutrition [26,56,57]. 

A higher level of education can in some cases provide people with greater access to information and knowledge about nutrition and its impact on health. While this may not always be the case and some highly educated people may adopt unhealthy dietary behaviors, this knowledge helps to make people more aware of the importance of a balanced diet, nutritional needs and the potential health risks associated with poor nutrition. On the other hand, it can also lead consumers to become more competent in interpreting nutrition labels, understanding dietary guidelines and critically evaluating nutrition information [58]. This enables them to make healthier food choices and adopt better eating habits.

On the other hand, higher education tends to develop critical thinking skills, which can sometimes help to question popular beliefs or to evaluate and analyze fad diets, leading people with more education to be less susceptible to influence. 

Regarding the income level variable, the data obtained show how women with a medium-high income level have a lower BMI and a higher IASE than those with a low-income level. The consumption of fast food and ultra-processed food is significantly lower, and the frequency of fish consumption is higher. There are no significant differences in the frequency of consumption of fried foods, or in the time spent on physical activity. 

This result seems to be in line with results described in previous studies showing that people with higher incomes tend to have access to healthier diets [59]. One possible explanation for this disparity in nutritional habits can be found in the affordability of healthier foods. Foods such as fresh fruits and vegetables, lean proteins and whole grains can sometimes be more expensive than processed or unhealthy food options [60]. People with higher incomes have more financial resources to afford these healthier foods and may face fewer constraints in purchasing nutritious ingredients. Generally, too, higher-income urban areas tend to have better access to supermarkets, farmers’ markets and specialized shops that offer a wide variety of fresh and nutritious foods. This compares with low-income neighborhoods that typically have limited access to healthy foods [61].

Another possible explanation relates to education which, as discussed above, is an important determinant of healthy food choices [56,57]. People with higher incomes tend to have more educational opportunities and may have a better understanding of nutrition and its impact on health.

Figure 1, which shows the PCA analysis of the nutrition and lifestyle variables, showing the color scale representing the contribution of the variables to the model, shows how the variables of frequency of water, coffee, and fish consumption, as well as a sedentary lifestyle, seem to provide little contribution and therefore do little to differentiate those subjects who adopt healthier behaviors from those who adopt less healthy habits. In future analyses, these variables could perhaps be disregarded because their discriminatory power among the subjects in the sample is low.

On the other hand, the variables in the frequency of consumption of fast food, fried food and ready meals have a high discriminatory power, making a relevant contribution to health.

The variables of alcohol consumption, IASE, BMI and minutes of weekly sports make a medium contribution.

If we look at the spatial arrangement of the variables, we see that the variables of fish and IASE are highly related and have the same direction, indicating that generally those people who have a high score on their healthy nutrition index consume fish frequently. The number of minutes per week devoted to physical activity is also related to the IASE and to fish consumption; people who do a lot of sport tend to have healthy nutritional habits. These habits also seem to influence their self-perceived level of health, as the corresponding arrow also points in the same direction.

In contrast, the consumption of fast food, ultra-processed food, and fried food is found to represent an angle of almost 180° with respect to the physical activity minutes variables, indicating an inverse relationship between the two groups of variables. Subjects with healthy physical activity habits tend to eat fast food, ultra-processed food, and fried food infrequently. BMI also has an inverse relationship with IASE and self-perceived health, indicating that those with low BMI tend to have a higher IASE and self-perceived health index.

Finally, there are groups of variables that form angles of around 90° between them, indicating that they are not related or that their relationship is weak. This is the case of BMI and alcohol with the frequency of consumption of fast food, ultra-processed food, and fried food.

If we reflect on the impact on health of the social and nutritional habits obtained from the sample, we can see that with respect to nutrition changes in diet are necessary; in fact, if we classify the IASE, with the same criteria followed in the study by Norte and Ortiz [37], on a 3 scale assigning 3 values to the IASE, on a scale of 3 values assigning the classification of ‘Healthy’ to those values of the nutritional index between 58.4 and 73, the classification of ‘Needs changes’ to those between 36.5 and 58.4 and ‘Unhealthy’ to those below 36.5, we see that for all the socio-demographic variables explored the average nutritional index obtained is in the range in which changes are necessary. 

Research shows that reducing sitting time can improve the metabolic consequences of obesity and improve the overall health of the individual. Sedentary behavior can be defined as individuals sitting for more than 6 h per day [20]. For the degree of sedentary behavior, it is therefore also recorded that changes need to be implemented given that for the sample explored the average time spent sitting is between 7 and 9 h. 

Regular physical activity provides health benefits and the prevention of many chronic diseases [19]. According to the Global Recommendations on Physical Activity for Health [62], 150 min of moderate aerobic activity or 75 min per week of vigorous aerobic physical activity is when the beneficial health effects begin. The mean value recorded for the sample, for each of the sociodemographic variables, suggests that the weekly time devoted to physical activity is insufficient, although we cannot conclude this definitively given that the survey did not discriminate between moderate and vigorous activity.

The consumption recommendation is ambiguous in most dietary guidelines [63]; in other research, alcohol does not nourish and instead contributes to weight gain [64]. Alcohol has been shown to affect the brain and most organs and systems and is associated with numerous health problems; therefore, it is advisable to limit its consumption in the diet. About alcohol consumption for all the socio-demographic variables explored, the average consumption is limited to values between two and four times a month, so this habit is healthy. 

In summary, most of the nutritional and lifestyle habits of the sample explored need improvement and this result could be contrasted with other evidence from the study such as BMI and the degree of self-perceived health. The BMI value is, in each of the socio-demographic ranges explored, within the normal weight range, contrary to what is seen in other studies on the Spanish adult population, where 53.7% of the population over 18 years of age suffers from obesity or overweight [65]. The feeling of general well-being is medium-high, as the degree of self-perceived health has presented a value between 3.65 and 3.90 out of 5 for each of the socio-demographic groups considered. This result could be explained by the young age of the sample considered. Younger individuals, on average, tend to have lower body mass index (BMI) and may experience better overall health compared to older individuals. In general, younger people may have higher metabolic rates, higher levels of physical activity, and fewer chronic health conditions compared to older individuals. 

We believe that the results obtained are interesting when planning a health intervention, given that they allow us to target the intervention more specifically to a certain population group and to certain habits with a greater probability of success.

### Study Strengths and Weaknesses

One of the weaknesses of the study conducted lies in the type of sampling used. Snowball sampling relies on participants recommending other potential participants, which can lead to a biased sample and limit control over the number of respondents. 

In our case, self-selection bias is observed in that several of the influencers and professionals who supported the dissemination of the survey were people concerned with and interested in nutrition and health. Consequently, the people who followed these networks and received the survey were also interested in these issues and sometimes had higher than average knowledge of the subject and healthier habits.

To try to minimize this bias, the survey was also disseminated through other channels, independent of the social networks, the main one being mailing to different associations and establishments carefully selected for covering the whole of Spain and for bringing together a heterogeneous public.

The large sample size and the coverage of the entire geography are undoubtedly the strengths of this study. A large sample size provides greater statistical power and reduces the survey’s margin of error. It allows for a more accurate representation of different demographic groups, which increases the likelihood that the findings are representative of the general population.

Reaching participants from different regions of Spain increases the external validity of the study. It helps ensure that the results reflect a more diverse range of perspectives and experiences, reducing the possibility of geographical bias. This geographical diversity allows for a more complete understanding of the topic under investigation and increases the applicability of the results to a wider population.

## 5. Conclusions

The results of the study show that most Spanish women need to make changes in their nutrition, as well as to lead a more active life, reducing their daily time spent sitting and increasing their weekly time dedicated to physical activity.

It has also been observed that adult women eat more healthily than younger women.

Finally, it is confirmed that a higher level of education and a higher socio-economic level are related to a better diet and healthier habits and, in general, to better health.

The field of interest of this study is very broad and this research may represent a starting point for future research. It could be of interest to analyze the lifestyle and nutrition of women of a higher age group and compare it successively with the results obtained in the present research to detect similarities and differences. It could also be interesting to be able to reproduce this study in other countries, adapting the instrument culturally and validating it beforehand, to compare the habits of the Spanish female population with those of the populations of other countries. Finally, given the importance that education seems to have in nutrition and health, future research, also multidisciplinary, could additionally focus on analyzing and developing effective training and awareness-raising methodologies in this field.

## Figures and Tables

**Table 1:** Variables of study.

Variables of Study			
General Data	Nutrition Habits	Physical Activity	Social Habits
Sex	Number of meals per day	Daily sitting time	Number of nights out
Age	Breakfast	Frequency of intense sport	Number of hours of sleep
Place of birth	Number of glasses of water	Duration of intense sport	Quality of sleep
Place of residence	Frequency of fruit consumption	Frequency of moderate sport	Sleep result
Size of municipality	Frequency of vegetable consumption	Duration of moderate sport	Ease of falling asleep
Work	Frequency of consumption of white and blue fish	Reason for lack of physical activity	Tobacco addiction
Level of education	Frequency of consumption of white and red meat		Frequency of alcohol consumption
Income level	Frequency of consumption of legumes		Quantity of alcohol consumed
Weight	Frequency of consumption of dairy products		Frequency of excessive alcohol consumption
Height	Frequency of cereal consumption		
Self-perceived level of health	Frequency of fast food consumption		
Illnesses requiring special diet	Frequency of consumption of fried food		
Symptoms of eating disorders	Frequency of ultra-processed food consumption		
Usual residence	Frequency of soft drinks consumption		
Origin survey	Frequency of fruit juice consumption		
	Frequency of consumption of coffee or energy drinks		
	Drinks to accompany meals		
	Special diet		

**Table 2:** Social and nutritional habits in relation to socio-economic variable groups.

	Mean ± SD or n (%)		
AGE	18–30 years	31–45 years	*p*-value *
Body mass index (BMI)	22.78 ± 4.00	24.06 ± 4.52	**4.06 × 10** **^−71^**
Healthy eating index (IASE)	53.19 ± 9.70	54.43 ± 9.84	**1.0686 × 10** **^−15^**
Self-perceived health	3.78 ± 0.81	3.80 ± 0.83	**0.0151**
Sedentary lifestyle	1.65 ± 0.86	1.53 ± 0.80	**4.85 × 10** **^−16^**
Physical activity (min)	148.32 ± 167.94	128.51 ± 150.12	**1.72 × 10** **^−9^**
Alcohol consumption	1.68 ± 0.77	1.67 ± 0.84	**0.0037**
Water consumption	3.41 ± 0.64	3.41 ± 0.63	0.8178
Coffee consumption	1.59 ± 0.65	1.75 ± 0.70	**1.53 × 10** **^−42^**
Fast-food consumption	2.53 ± 0.74	2.35 ± 0.76	**3.81 × 10** **^−38^**
Consumption of fried foods	2.35 ± 0.83	2.13 ± 0.78	**7.76 × 10** **^−55^**
Consumption of ultra-processed food	2.48 ± 0.93	2.35 ± 0.95	**1.41 × 10** **^−15^**
Fish consumption	1.75 ± 0.51	1.82 ± 0.47	**1.47 × 10** **^−15^**
**EDUCATIONAL LEVEL**	**Basic studies**	**Superior studies**	***p*** -value *
Body mass index (BMI)	24 ± 4.77	23.15 ± 4.08	**6.59 × 10** **^−16^**
Healthy eating index (IASE)	51.95 ± 10.29	54.56 ± 9.47	**8.38 × 10** **^−43^**
Self-perceived health	3.65 ± 0.87	3.85 ± 0.79	**3.02 × 10** **^−34^**
Sedentary lifestyle	1.52 ± 0.80	1.62 ± 0.84	**7.95 × 10** **^−13^**
Physical activity (min)	134.32 ± 170.60	140.51 ± 155.11	**3.01 × 10** **^−9^**
Alcohol consumption	1.58 ± 0.77	1.71 ± 0.	**2.95 × 10** **^−20^**
Water consumption	3.41 ± 0.67	3.42 ± 0.62	0.5632
Coffee consumption	1.63 ± 0.70	1.69 ± 0.67	**4.63 × 10** **^−9^**
Fast-food consumption	2.50 ± 0.76	2.42 ± 0.75	**1.75 × 10** **^−7^**
Consumption of fried foods	2.36 ± 0.86	2.19 ± 0.79	**5.41 × 10** **^−24^**
Consumption of ultra-processed food	2.52 ± 0.95	2.37 ± 0.93	**1.59 × 10** **^−14^**
Fish consumption	1.72 ± 0.51	1.81 ± 0.48	**1.78 × 10** **^−21^**
**INCOMES LEVEL**	**Low Incomes**	**High Incomes**	***p*** -value *
Body mass index (BMI)	23.65 ± 4.48	23.13 ± 4.10	**1.72 × 10** **^−9^**
Healthy eating index (IASE)	52.85 ± 10.03	54.81 ± 9.41	**2.39 × 10** **^−30^**
Self-perceived health	3.69 ± 0.84	3.90 ± 0.77	2.26 × 10^−17^
Sedentary lifestyle	1.54 ± 0.82	1.64 ± 0.84	**0.000**
Physical activity (min)	142.90 ± 166.37	134.17 ± 152.35	**0.30**
Alcohol consumption	1.64 ± 0.78	1.72 ± 0.82	**2.34 × 10** **^−9^**
Water consumption	3.41 ± 0.64	3.42 ± 0.62	0.68
Coffee consumption	1.64 ± 0.69	1.69 ± 0.68	**3.41 × 10** **^−7^**
Fast-food consumption	2.47 ± 0.76	2.42 ± 0.74	0.0005
Consumption of fried foods	2.25 ± 0.84	2.23 ± 0.78	**0.33**
Consumption of ultra-processed food	2.45 ± 0.95	2.38 ± 0.93	9.99 × 10^−6^
Fish consumption	1.75 ± 0.51	1.82 ± 0.48	**6.05 × 10** **^−19^**

* Mann–Whitney test.

**Figure A4:**
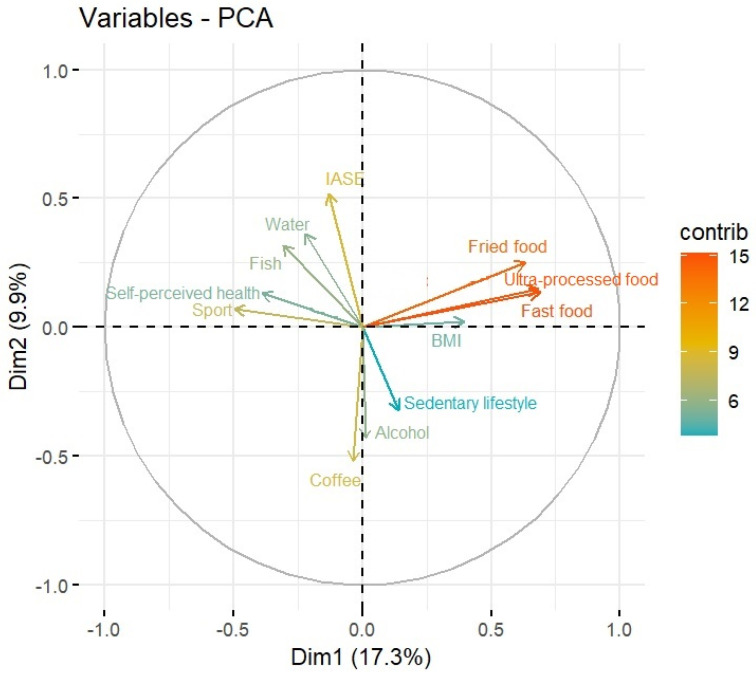
Graphical representation of the PCA models in 2 dimensions of population between 31 and 45 years.

## Data Availability

Not applicable.

## Appendix B

A PCA model was done to study the relationships between the variables in the adult population (31–45 years). This model explains the 27.2% of data variance with two components: the first explains the 17.3% and the second one, the 9.9%. We have selected this number of components attending the scree plot: the percentage variability explained by a new principal component is almost the same as the previous one. In addition, a PCA model’s interpretability is more straightforward, considering two dimensions than three or more.

The first dimension is mainly explained by Fast Food, Ultra-processed Food, Fried Food, and Sport. On the other hand, Coffee, IASE, Alcohol, and Water are the variables that contribute the most to the second dimension.

Regarding the variables plot (Figure A4), Fish and Self-perceived health seems to be correlated, as the arrows are nearby. However, the contribution of these two variables is not extremely high. Additionally, the variables plot is pretty similar to Figure 1.
